# Effects of anti-inflammatory dietary patterns on non-alcoholic fatty liver disease: a systematic literature review

**DOI:** 10.1007/s00394-023-03085-0

**Published:** 2023-01-24

**Authors:** Jayden Abdallah, Samantha Assaf, Arpita Das, Vasant Hirani

**Affiliations:** 1 Discipline of Nutrition and Dietetics, Charles Perkins Centre, The University of Sydney, Sydney, Australia; 2 The University of Sydney, The Concord Health and Ageing Men Project, Camperdown, Australia

**Keywords:** NAFLD, Fatty liver, Hepatic steatosis, Mediterranean diet, Inflammation

## Abstract

**Purpose:**

Non-alcoholic fatty liver disease (NAFLD) is the leading chronic hepatic condition. Low-grade chronic inflammation contributes to disease progression. Diet has protective effects on hepatic health and inflammatory pathways. The purpose of this review is to systematically review and describe the effects of anti-inflammatory dietary patterns on NAFLD.

**Methods:**

The Cochrane CENTRAL Library, Cumulative Index of Nursing and Allied Health Literature, Embase, MEDLINE and Web of Science databases were searched. A total of 252 records were identified, 7 of which were included in this review. The revised Cochrane risk-of-bias tool was used to conduct a quality assessment for randomised trials. Certainty of evidence was assessed using the grading of recommendations, assessment, development, and evaluation tool.

**Results:**

Of the 7 included studies, 6 were classified as low risk of bias and studies ranged from high to very low certainty of evidence. In the randomised-controlled studies systematically reviewed, either adherence to the Mediterranean, DASH, or FLiO diet was studied, against usual care or energy matched controls, with a total of 255 participants. Anti-inflammatory dietary pattern adherence significantly reduced the severity of most hepatic and inflammatory markers, and secondary outcomes. A minority of outcomes were improved significantly more than controls.

**Conclusion:**

Anti-inflammatory dietary patterns showed benefits to NAFLD risk factors, severity markers and inflammatory markers compared to the control diet. It is unclear whether reductions in the evaluated parameters are related solely to the anti-inflammatory diet or weight loss resulting from caloric restriction, as improvements in control groups were also evidenced. Current limited body of evidence indicates need for further research including isocaloric dietary patterns, longer interventions, measures of inflammatory markers, and studies including normal-weight subjects to confirm findings at higher certainty.

**PROSPERO Registration:**

CRD42021269382.

**Supplementary Information:**

The online version contains supplementary material available at 10.1007/s00394-023-03085-0.

## Introduction

In Western countries, non-alcoholic fatty liver disease (NAFLD) is the most common chronic liver disease [1]. Risk factors for NAFLD cause low-grade chronic inflammation which contributes to progression towards higher risk cirrhotic states [2]. Dietary intake of nutrients also has effects on inflammatory pathways within the body whilst many sources of pro-inflammatory compounds are comprised within foods common to the diet of large populations [3]. With current recommendations indicating the most effective treatment is weight reduction through lifestyle modifications [4], it is important to study what dietary modifications are most effective. Increased consumption of foods with high energy density and sugar sweetened beverages are key mediators of obesity, oxidative stress, and related hepatic lipid influx [5]. Current studies suggest there is an association between liver health and nutrition with therapeutic intervention strategies commonly involving modification of dietary intake. Given there is a clear correlation between NAFLD and inflammation, it is important to assess how that is affected by changes in dietary habits, more specifically, the adherence of anti-inflammatory diets.

A growing awareness of the health benefits of anti-inflammatory dietary patterns is reflected in the literature, yet a specific dietary definition is yet to exist. Instead, it is characterised by a set of eating patterns with anti-inflammatory properties from nutrients found with increased intake of vegetables, legumes, whole grains, fruits, fish, and olive oil whilst reducing intake of red meat, dairy, and refined carbohydrates, as is foundational to the Mediterranean diet and the Dietary Approaches to Stop Hypertension (DASH) diet [3, 4]. These diets are indicated to be an effective therapeutic option because of its beneficial effects on all risk factors associated with metabolic syndrome and NAFLD development, offering protective anti-inflammatory properties.

There are proven anti-inflammatory effects of the Mediterranean diet as a whole [6], and its components, such as olive oil [7–9] and nuts [10]. The Mediterranean diet has been proven to have protective benefits against cardiovascular disease (CVD) [4], and studies have shown an association between NAFLD and CVD, possibly due to similar risk factors [11]. The DASH diet has shown improvements in circulating serum inflammatory biomarkers in adults, when compared to their usual diet, proving it to be a valuable strategy in suppressing inflammatory processes [12]. Within these diets, common components including low GI and fibre rich-legumes has been found to significantly improve hs-CRP levels [13, 14] and reduce inflammatory processes by suppressing the production of inflammatory cytokines [15–17]. In addition, antioxidant compounds in fruits and vegetables [18] may also have anti-inflammatory properties [19–21].

There are no other systematic reviews identified that examine the relationship between anti-inflammatory dietary patterns and inflammatory markers, as well as NAFLD progression and severity. Therefore, the aim of this research is to systematically review the literature and describe the effects of anti-inflammatory dietary patterns on non-alcoholic fatty liver disease in adults aged 18 years and over.

## Methods

Prior to study commencement, the systematic review was registered at the PROSPERO International Prospective Register of Systematic Reviews (CRD42021269382) to avoid duplication and promote transparency [22] and was reported following the PRISMA (Preferred Reporting Items for Systematic Reviews and Meta-Analyses) guidelines (Table S1).

### Database search

To find relevant and existing literature, a comprehensive database search was conducted by two reviewers for records available prior to the 16th of September 2021. Electronic databases used included Cochrane CENTRAL Library, Cumulative Index of Nursing and Allied Health Literature (CINAHL), Embase, MEDLINE, and Web of Science.

In order to conduct extensive research, search strategy key terms were separated into three groups: population, exposure and outcome of interest, and are as follows: “non-alcoholic fatty liver disease”, “fatty liver, non-alcoholic”, “fatty livers, non-alcoholic”, “liver, non-alcoholic fatty”, “livers, non-alcoholic fatty”, “non-alcoholic fatty liver”, “non-alcoholic fatty livers”, “non-alcoholic steatohepatitis”, “steatohepatitis, non-alcoholic” for population and search terms; and “inflammatory dietary pattern”, OR “dietary patterns”, OR “low Inflammatory diet”, OR “pro-inflammatory diet”, OR “anti-Inflammatory diet”, OR “anti- Inflammatory dietary pattern”, OR “anti-Inflammatory type diet”, OR “dietary inflammatory index” OR “DII”, OR “D-AII”, OR “Mediterranean diet”, OR “Mediterranean dietary pattern”, OR “Mediterranean type diet”, OR “DASH diet” AND “inflammation” (“inflammation”, “cytokines” OR “c-reactive protein”, OR “crp”, “interleukin”, “IL-6”, “tumour necrosis factor”, “tnf”, “adiponectin”, “fibrinogen”).

### Screening and selection criteria

Following the database search, the first stage of screening involved assessing titles and abstracts against inclusion and exclusion criteria (Table 1), by two reviewers JA and SA. From this, the journal articles that were selected underwent a second stage of screening via full-text retrieval by the same two reviewers, using the same criteria.

**Table 1:** Inclusion and exclusion criteria

Parameter	Inclusion criteria	Exclusion criteria
Population	1. Individuals aged ≥ 18 years with NAFLD confirmed	1. Individuals aged < 18 years and with alcohol-induced hepatic steatosis, with advanced chronic liver disease such as cirrhosis, and using enteral or parenteral nutrition
		2. Mixed-population studies that included, in addition to patients with NAFLD, healthy individuals or those with diseases not separated by groups
		3. Animal studies
Intervention or exposure	1. Interventions of anti-inflammatory dietary patterns including all mode of deliveries such as direct meal deliveries and dietary advice provided by trained professionals	1. Anti-inflammatory dietary patterns supplemented with commercial supplements
	2. Interventions involving anti-inflammatory dietary patterns with the manipulation of nutrient composition with a whole diet approach or dietary patterns supplemented with food items	2. Studies including other interventions in addition to anti-inflammatory dietary patterns
	3. Exposures to pro-inflammatory dietary patterns assessed	
Comparison	1. The inactive control diet (such as a placebo, no treatment, usual care without dietary advice or a waiting list control)	Not applicable
	2. Comparator diet	
Outcomes	1. NAFLD, hepatic steatosis attenuation, ALT and AST levels	1. Alcohol-induced hepatic steatosis, other chronic liver diseases such as cirrhosis
		2. Parameters expressed qualitatively
Study design	1. RCTs, cluster RCTs, pseudo-randomised-controlled trial, non-randomised-controlled clinical trials, cluster trials, prospective cohort studies, retrospective cohort studies	1. Controlled before-after studies, ITS studies without a control group, cross-sectional studies, case series, case reports, non-study-based sources, narrative reviews and systematic reviews
		2. Interrupted time-series studies with a control group, case–control studies, cross-sectional studies and nested case–control studies
Language	English	Other than English

### Data extraction

Data were extracted in duplicates from each study by two reviewers (SA, JA) and the extracted data were checked by the same two reviewers. Data extracted includes study details (author, year of publication, study’s country, study design, setting, recruitment, eligibility criteria, and follow-up duration), population characteristics (age, sex, race, sample size, withdrawal or exclusions, and underlying disease status of participants), intervention or exposure (dietary pattern studied, diet assessment method, level of dietary control, randomisation, and comparator), and outcomes (hepatic markers, inflammatory markers, anthropometric markers, glycaemic markers, and lipid markers), statistical method and potential confounders. The authors were contacted for any missing full text or data.

### Quality assessment

A quality assessment was conducted by JA and SA, independently, to assess the risk of bias using the revised Cochrane risk-of-bias tool for randomised trials (RoB 2.0) at the study level. From this, they were rated as ‘low risk of bias’, “high risk of bias” or “some concerns”. The outcome as effect estimates by dietary pattern of each study were assessed for certainty using GRADE (Grading of Recommendations Assessment, Development and Evaluation) and were categorised appropriately as one of the following: “high”, “moderate”, “low”, “very low”.

## Results

The search strategy retrieved 252 studies from the accessed databases and registers; 60 were excluded for cross-database duplication screening. After reviewing the titles and abstracts, 162 publications were excluded, and 30 articles were eligible for full-text retrieval; however, one article could not be retrieved due to availability. After carefully assessing the 29 articles, 25 were excluded, leaving 4 studies. A further 4 articles were retrieved following citation searching within these articles, with 1 excluded. A final 7 studies were included to be systematically reviewed.

**Fig. 1:**
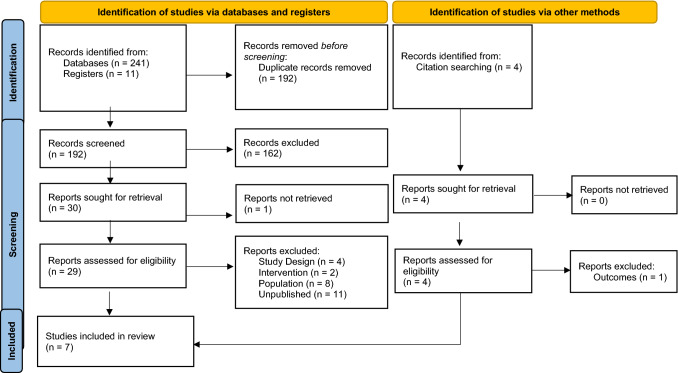
Flowchart presenting article selection process. [42]. From: Page MJ, McKenzie JE, Bossuyt PM, Boutron I, Hoffmann TC, Mulrow CD, et al. The PRISMA 2020 statement: an updated guideline for reporting systematic reviews. BMJ 2021;372:n71. https://doi.org/10.1136/bmj.n71. For more information, visit:http://www.prisma-statement.org/

A schematic diagram of the search strategy and reasons for exclusions are presented in Fig. 1. The duration of the intervention studies ranged from 6 weeks to 24 months; the participants were adults aged 18 and above; and the sample size ranged from 12 to 98 participants. In this review, the data are from 255 adults, 131 were part of the intervention group and 136 were part of the control group with studies conducted in Greece (1), Spain (2), Australia (2), Iran (1), and Serbia (1). Study characteristics are presented (Table 2).

**Table 2:** Characteristics of studies systematically reviewed

Reference; study design	Sample size	Characteristics	Outcome measurements	Quality assessment (Cochrane RoB 2.0)
Katsagoni et al. (2018) [23] Randomised-controlled trial Greece	Final sample: 35/42 (83% participation) Excluded/withdrew: 7	Age range: 18–65 years % Sex (F/M): 38/62	Ultrasonography, anthropometric measures, blood analysis	Low risk of bias
Marin-Alejandre et al. (2019) [24] Randomised-controlled trial Spain	Final sample: 76/98 (78% participation) Excluded/withdrew: 22	Mean age: 50 years % Sex (F/M): 48/52	Ultrasonography, anthropometric measures, blood analysis, MRI	Low risk of bias
Marin-Alejandre et al. (2021) [25] Randomised-controlled trial Spain	Final sample: 58/98 (59% Participation) Excluded/withdrew: 40	Mean age: 50 years % Sex (F/M): 48/52	Ultrasonography, anthropometric measures, blood analysis, MRI	Low risk of bias
Properzi et al. (2018) [26] Randomised-controlled trial Australia	Final sample: 48/51 (94% participation) Excluded/withdrew: 3	Mean age: 52 years % Sex (F/M): 49/51	Ultrasonography, anthropometric measures, blood analysis, MRS	Low risk of bias
Razavi Zade et al. (2016) [27] Randomised-controlled trial Iran	Final sample: 60/75 (80% participation) Excluded/withdrew: 15	Mean age: 41 years % Sex (F/M): 50/50	Ultrasound, anthropometric measures, blood analysis	Low risk of bias
Ristic-Medic et al. (2020) [28] Randomised-controlled trial Serbia	Final sample: 24/27 (89% participation) Excluded/withdrew: 3	Mean age: 34 years % Sex (F/M): 0/100	Ultrasonography, anthropometric measures, blood analysis	Low risk of bias
Ryan et al. (2013) [29] Crossover randomised-controlled trial Australia	Final sample: 12/12 (100% participation) Excluded/withdrew: 0	Mean age: 55 years % Sex (F/M): 50/50	Ultrasonography, MRI, H-MRS, blood analysis and anthropometric measures	Some concerns

The interventions involved the Mediterranean diet (MD) in four studies [23, 26, 28, 29], Fatty Liver in Obesity (FLiO) diet in two studies [24, 25] and Dietary Approaches to Stop Hypertension (DASH) diet each in one study [27]. Comparison dietary interventions included low-fat (LF) diet [26, 28], low-fat high carbohydrate (LFHC) diet [29], American Heart Association (AHA) [24, 25], and macronutrient equivalents to anti-inflammatory dietary patterns [23, 27]. Inflammatory and anti-inflammatory markers were measured in 4 studies [24, 25, 27, 28]. Hepatic steatosis, as liver fat, raw hepatic fat, or intra-hepatic lipid %, was measured with magnetic resonance spectroscopy or ultrasound in four studies [24–26, 29]. NAFLD grade was measured in one study [27]. Liver stiffness was measured using shear wave elastography in four studies [23, 24, 26], FLI and HSI were measured in three studies [24, 25, 28]. Varying serum liver enzyme levels were evaluated in all studies [23–29].

### Risk of bias analysis

A risk-of-bias assessment was conducted using the Cochrane RoB 2 tool for randomised-controlled trials and cross-over randomised-controlled trials (Table S2). All studies were classified as being at “low risk of bias” in all evaluated items. This is except for Ryan et al. 2013 which was classified as having “some concerns” for its final judgement as well as “some concerns” within the domain relevant to cross-over RCTs of risk of bias arising from period and carryover effects (Domain S) [29].

### Primary outcomes

A detailed review of outcomes measured in studies of interest is provided, where only statistically significant results are reported (Table 3). The two studies measuring inflammatory markers reported a statistically significant reduction in hs-CRP with MD and DASH interventions [27, 28]. There was no significant difference between MD and LF [28]. Regarding markers of oxidative stress, a significant improvement was reported in glutathione (GSH) and malondialdehyde (MDA) but not total antioxidant capacity (TAC) with DASH [27]. There was a greater reduction in NAFLD with the DASH intervention compared with control [27]. Hepatic steatosis was significantly reduced in two studies with MD intervention, but not with significant difference to the LF control group [26, 29]. The two studies with FLiO interventions also significantly reduced hepatic steatosis, with no significant difference between AHA control [24, 25]. In these studies, liver stiffness was significantly reduced only 24 months with the FLiO intervention [24, 25]. In two studies incorporating an MD, significant improvement in liver stiffness was noted in one of these 6-month interventions [25], but not the other [26]. The three studies measuring FLI and HSI showed a significant decrease with MD and FLiO interventions, and AHA and LF control groups [24, 25, 28]. All studies evaluated the impact of dietary intervention on ALT, with all but Ryan et al. (2013) demonstrating a significant improvement [29]. There was no significant reduction in ALT when compared with a control group except Razavi Zade et al. (2016) [27]. A significant reduction was noted in all four studies measuring AST with MD, DASH, AHA and FLiO interventions [24, 25, 27, 28], and there was no significant difference to the LF control [28]. Three studies reported significant reduction in GGT with MD interventions, of which Ristic-Medic et al. (2020) reported a significant reduction in GGT with the LF control, whereas Properzi et al. (2018) and Katsagoni et al. (2018) did not [23, 26, 28]. Marin-Alejandre et al. and Ryan et al. (2013) reported no significant change in GGT with interventions; however, Marin-Alejandre et al. (2019) reported a significant reduction with AHA control at 6 and 12 months [24, 25, 29]. Razavi Zade et al. uniquely measured grade of fatty liver where it was reported that all patients achieved a significant reduction in grade with the DASH diet [27].

**Table 3:** Extracted data from studies systematically reviewed

Article	Results			Summary
Katsagoni et al. (2018) [23]	Baseline → 6 months	MD (energy restricted)	Control (energy/macronutrient equivalent)	In a 6-month study on the effects of a Mediterranean diet and lifestyle weight loss programme in patients with NAFLD, Mediterranean diet significantly improved liver stiffness, BMI, LDL and non-HDL cholesterol but did not improve other liver function outcomes compared to the control group. All other secondary outcomes showed no significant difference after the programme
	Primary			
	ALT (U/I)	51 (30–73) → 34 (24–46)^‡^	44 (24–64) → 44 (32–58)^‡^	
	GGT (U/I)	65 (28–96) → 40 (23–64)^‡^	64 (34–170) → 73 (31–166)^‡^	
	Liver stiffness (kPa)^§^	6.6 (5.5–9.8) → 6.2 (5.1–9.9)^‡^	7.8 (5.5–10.9) → 8.0 (6.1–11.1)^‡^	
	NAFLD fibrosis score	− 2.36 ± 1.3 → − 2.38 ± 1.5^‡^	− 2.19 ± 1.4 → -2.21 ± 1.1^‡^	
	Secondary			
	Weight (kg)	96.7 (79.6–101) → 83.0 (75.3–98.0)^‡^	89.3 (79.4–95.2) → 86.4 (78.4–95.0)^‡^	
	BMI (kg/m^2^)	31.67(27.4–33.6) → 28.21(25.9–31.5)^‡^	30.04 (28.2–33.1) → 29.49 (27.8–32.9)^‡^	
	Abnormal WC (n)	76.2 (16%) → 47.6 (10%)^‡^	90.5 (19%) → 76.2 (16%)^‡^	
	HOMA-IR	3.4 (1.9–5.1) → 2.6 (1.7–4.1)^‡^	2.5 (1.7–4.0) → 2.5 (1.4–3.6)^‡^	
	TC (mmol/L)	5.1 ± 1.3 → 4.8 ± 1.2^‡^	5.3 ± 1.0 → 5.6 ± 1.2 ^‡^	
	HDL (mmol/L)	1.0 (0.9–1.5) → 1.1 (1.0–1.6)^‡^	1.1 (0.9–1.5) → 1.1 (0.9–1.6)^‡^	
	LDL (mmol/L)^§^	3.2 ± 1.0 → 2.9 ± 0.9^‡^	3.3 ± 0.9 → 3.6 ± 1.0^‡^	
Marin-Alejandre et al. (2019) [24]	Baseline → 6 months	FLiO diet (energy restricted)	AHA diet (energy restricted)	In patients with NAFLD and overweight or obesity, both calorie-restricted AHA and FLiO diets improved primary and secondary outcomes at 6 months. The FLiO diet provided greater benefit to primary outcomes; however, both diets had a similar effect on secondary outcomes. The FLiO group maintained a significant decrease in ALT, liver fat, TE liver stiffness at 24 months compared to baseline values, whilst no significant changes were observed in the AHA group after 24 months. There was no difference in hepatic parameters between groups when adjusted for weight loss
	Primary			
	AST (IU/L)		25.5 ± 11 → 21.6 ± 6	
	ALT (IU/L)	33.3 ± 18 → 21.7 ± 9	33.1 ± 17 → 22.9 ± 9	
	GGT (IU/L)		40.9 ± 29 → 28.3 ± 23	
	Steatosis degree	1.5 ± 0.6 → 0.7 ± 0.6	1.5 ± 0.7 → 0.8 ± 0.7	
	Liver fat (%)	7.0 ± 5 → 2.8 ± 3	7.4 ± 5 → 3.8 ± 3	
	TE liver stiffness (kPa)			
	FLI	76.9 ± 21 → 47.9 ± 24	80.4 ± 16 → 54.4 ± 24	
	Adiponectin (μg/mL)^§^	6.6 ± 2 → 9.5 ± 4	6.7 ± 2 → 8.0 ± 3	
	HSI	45.2 ± 5 → 39.1 ± 4	45.1 ± 5 → 39.9 ± 5	
	Secondary			
	Weight (kg)	95.1 ± 14 → 86.6 ± 13	94.4 ± 14 → 84.2 ± 13	
	BMI (kg/m^2^)	33.3 ± 4 → 30.1 ± 4	33.7 ± 4 → 30.2 ± 4	
	WC (cm)	108 ± 9 → 99 ± 10	110 ± 10 → 99 ± 15	
	TC (mg/dL)	197 ± 35 → 185 ± 41	192 ± 40 → 177 ± 43	
	HDL-C (mg/dL)			
	LDL-C (mg/dL)			
	Triglycerides (mg/dL)	129 ± 62 → 91 ± 59	129 ± 66 → 99 ± 41	
	HOMA-IR	4.2 ± 2 → 2.6 ± 2	4.6 ± 3 → 2.7 ± 2	
Marin-Alejandre et al. (2021) [25]	Baseline → 12 months			
	Primary			
	AST (IU/L)^§^	23.9 ± 8 → 20.6 ± 8	25.5 ± 11 → 25.1 ± 9*	
	ALT (IU/L)	33.3 ± 18 → 24.4 ± 13	33.1 ± 17 → 25.7 ± 11	
	GGT (IU/L)		40.9 ± 29 → 31.4 ± 20	
	Steatosis degree	1.5 ± 0.6 → 0.6 ± 0.7	1.5 ± 0.7 → 1.0 ± 0.9	
	Liver fat (%)	7.0 ± 5 → 5.6 ± 6	7.4 ± 5 → 5.1 ± 4	
	TE liver stiffness (kPa)		5.2 ± 2 → 4.4 ± 1	
	FLI§	76.9 ± 21 → 51.0 ± 26	80.4 ± 16 → 62.7 ± 24	
	Adiponectin (μg/mL)	6.6 ± 2 → 8.8 ± 3	6.7 ± 2 → 8.3 ± 4	
	HSI	45.2 ± 5 → 40.5 ± 5	45.1 ± 5 → 40.7 ± 7	
	Secondary			
	Weight (kg)	95.1 ± 14 → 86.3 ± 13	94.4 ± 14 → 87.3 ± 15	
	BMI (kg/m^2^)	33.3 ± 4 → 30.1 ± 4	33.7 ± 4 → 31.2 ± 5	
	WC (cm)	108 ± 9 → 98 ± 10	110 ± 10 → 102 ± 11	
	TC (mg/dL)	197 ± 35 → 180 ± 34		
	HDL-C (mg/dL)	53.8 ± 12 → 57.3 ± 13		
	LDL-C (mg/dL)	118 ± 30 → 104 ± 29		
	Triglycerides (mg/dL)^§^	129 ± 62 → 93 ± 41	129 ± 66 → 117 ± 49*	
	HOMA-IR	4.2 ± 2 → 3.0 ± 3	4.6 ± 3 → 3.3 ± 2	
	Baseline → 24 months			
	Primary			
	AST (IU/L)			
	ALT (IU/L)^§^	33.3 ± 18 → 21.8 ± 7	33.1 ± 17 → 25.7 ± 11*	
	GGT (IU/L)			
	Steatosis degree	1.5 ± 0.6 → 0.5 ± 0.6	1.5 ± 0.7 → 0.9 ± 0.9	
	Liver fat (%)	7.0 ± 5 → 4.5 ± 3		
	TE liver stiffness (kPa)^§^	4.7 ± 2 → 3.7 ± 1	5.2 ± 2 → 4.4 ± 1*	
	FLI^§^	76.9 ± 21 → 56.8 ± 27	80.4 ± 16 → 69.2 ± 26	
	Adiponectin (μg/mL)^§^	6.6 ± 2 → 10.6 ± 3	6.7 ± 2 → 8.4 ± 3	
	HSI	45.2 ± 5 → 39.7 ± 5	45.1 ± 5 → 42.4 ± 7	
	Secondary			
	Weight (kg)	95.1 ± 14 → 89.1 ± 13	94.4 ± 14 → 89.8 ± 16	
	BMI (kg/m^2^)	33.3 ± 4 → 30.8 ± 4	33.7 ± 4 → 32.1 ± 5	
	WC (cm)^§^	108 ± 9 → 102 ± 11	110 ± 10 → 108 ± 13*	
	TC (mg/dL)			
	HDL-C (mg/dL)^§^	53.8 ± 12 → 57.3 ± 13*	51.9 ± 14 → 52.8 ± 14*	
	LDL-C (mg/dL)			
	Triglycerides (mg/dL)			
	HOMA-IR	4.2 ± 2 → 2.6 ± 2	4.6 ± 3 → 3.4 ± 2	
Properzi et al. (2018) [26]	Baseline → 12 weeks	MD	LF diet	There was no significant difference in hepatic steatosis between ad libitum Mediterranean or low-fat diet over 12 weeks. Both diets led to a similar degree of reduction in hepatic steatosis (and resolution of NAFLD. The Mediterranean diet significantly reduced ALT, GGT, raw hepatic fat, body weight, waist circumference, BMI, total cholesterol and HbA1c
	Primary			
	ALT (IU/L)	77 ± 51 → 69 ± 47	68 ± 66 → 56 ± 45	
	GGT (IU/L)	102 ± 120 → 83 ± 99		
	Raw hepatic fat (%)	34.2 ± 16.3 → 24.0 ± 14.7		
	HepaScore			
	Liver stiffness (kPa)			
	Secondary			
	Weight (kg)	89.3 ± 12.7 → 87.3 ± 12.5	81.3 ± 13.3 → 79.6 ± 13.5	
	WC (cm)§	105.6 ± 10.3 → 102.9 ± 10.4	98.0 ± 12.0 → 93.9 ± 10.6	
	BMI (kg/m^2^)	31.8 ± 4.0 → 31.1 ± 4.0	30.1 ± 5.69 → 29.5 ± 5.8	
	TC (mg/dL)	184.8 ± 49.9 → 175.2 ± 49.5		
	Triglyceride (mg/dL)	165.6 ± 76.2 → 144.2 ± 76.2		
	HDL-C (mg/dL)			
	LDL-C (mg/dL)			
	HOMA-IR		2.76 ± 1.52 → 2.95 ± 4.32	
Razavi Zade et al. (2016) [27]	Baseline → 8 weeks	DASH diet (energy restricted)	Control (energy/macronutrient equivalent)	In patients with NAFLD and overweight or obesity, adherence to a calorie-restricted DASH diet for 8 weeks showed significant beneficial effect on serum liver enzymes, inflammatory markers, anthropometric indices, markers of insulin metabolism, cholesterol, and triglycerides. A greater percentage of patients in the DASH group decreased NAFLD grade than control
	Primary			
	AST (IU/L)	42.7 ± 34.1 → 32.0 ± 16.6		
	ALT (IU/L)^§^	36.4 ± 19.1 → 28.0 ± 20.8		
	ALP (IU/L)^§^	206.2 ± 54.8 → 179.9 ± 55.9		
	hs-CRP (ng/mL)^§^	4823.1 ± 3358.9 → 3598.4 ± 2752.6	4957.0 ± 3421.8 → 4637.5 ± 2872.0*	
	TAC (mmol/L			
	GSH (μmol/L)^§^	585.0 ± 78.8 → 652.8 ± 144.4	586.1 ± 81.6 → 590.6 ± 93.1*	
	MDA (μmol/L)^§^	3.5 ± 0.6 → 3.2 ± 0.3	3.2 ± 0.4 → 3.1 ± 0.5*	
	NAFLD Grade I (%)	20.0 → 66.7	26.7 → 63.3	
	NAFLD Grade II (%)	46.7 → 33.3	46.7 → 16.7	
	NAFLD Grade III (%)	33.3 → 0.0	26.7 → 20.0	
	Secondary			
	WC (cm)^§^	99.3 ± 8.4 → 95.1 ± 7.7	94.9 ± 12.7 → 92.3 ± 12.2	
	HOMA-IR§	2.9 ± 1.5 → 2.1 ± 1.4		
	VLDL-C (mg/dL)^§^	32.9 ± 12.9 → 26.6 ± 10.7	31.6 ± 11.6 → 31.7 ± 19.3*	
	TC (mg/dL)	187.4 ± 33.9 → 173.4 ± 33.0		
	LDL-C (mg/dL)			
	HDL-C (mg/dL)	42.7 ± 5.8 → 46.1 ± 6.0		
	Triglycerides (mg/dL)^§^	164.3 ± 64.5 → 133.0 ± 53.7	158.1 ± 58.2 → 158.4 ± 96.7*	
Ristic-Medic et al. (2020) [28]	Baseline → 12 weeks	MD (energy restricted)	LF diet (energy restricted)	In patients with NAFLD and overweight or obesity, the calorie-restricted Mediterranean and low-fat diet both significantly improved lipid profiles, liver indices, cholesterol, glucose, insulin, and anthropometry. The Mediterranean diet decreased FLI greater than the low-fat diet
	Primary			
	AST (IU/L)^§^	32.50 (23.00–32.75) → 20.00 (16.00–21.75)	35.65 (25–41.50) → 25.50 (18.75–30.75)	
	ALT (IU/L)	65.33 ± 23.90 → 27.33 ± 6.46	63.17 ± 15.76 → 31.92 ± 11.89	
	GGT (IU/L)	47.42 ± 36.25 → 24.33 ± 11.57	42.53 ± 10.48 → 27.08 ± 9.90	
	hs-CRP (mg/L)	1.02 (0.75–2.23) → 0.81 (0.34–1.40)	2.10 (0.98–3.20) → 0.77 (0.54–1.27)	
	FLI§	81.92 ± 9.95 → 43.17 ± 7.99	83.52 ± 10.76 → 55.08 ± 18.22	
	HSI	47.6 ± 4.92 → 39.34 ± 3.24	45.85 ± 4.63 → 37.67 ± 4.07	
	Secondary			
	Weight (kg)	101.11 ± 9.09 → 91.88 ± 9.48	102.12 ± 8.19 → 92.41 ± 8.14	
	BMI (kg/m^2^)	30.43 ± 1.81 → 27.65 ± 1.80	30.17 ± 2.28 → 27.68 ± 2.44	
	WC (cm)	105.67 ± 5.94 → 95.83 ± 5.73	107.58 ± 6.96 → 98.83 ± 8.04	
	Triglycerides (mmol/L)^§^	1.92 (1.35–2.55) → 1.06 (0.80–1.20)	2.40 (1.55–2.69) → 1.29 (1.20–1.57)	
	TC (mmol/L)	6.00 ± 0.78 → 4.83 ± 0.95	6.08 ± 0.69 → 4.81 ± 0.82	
	LDL-C (mmol/L)	3.67 ± 0.72 → 2.88 ± 0.82	3.96 ± 0.89 → 3.14 ± 0.85	
	HDL-C (mmol/L)	1.29 ± 0.13 → 1.41 ± 0.15	1.22 ± 0.12 → 1.28 ± 0.11	
	HOMA-IR	3.96 (3.40–4.76) → 2.63 (2.28–3.04)	4.07 (3.45–44.43) → 2.86 (2.53–3.37)	
Ryan et al. (2013) [29]	Baseline → 6 weeks	MD	LFHC diet	In subjects on a Mediterranean diet for 6 weeks, there was a significant decrease in intra-hepatic lipid, and insulin. No other significant benefit on both primary and secondary outcomes was reported. In comparison, the control group did not show any significant differences in primary or secondary outcomes
	Primary			
	ALT (IU/L)			
	GGT (IU/L)			
	Intra-hepatic lipid (%)	14.2 ± 11.7 → 8.6 ± 7.0		
	Secondary			
	Weight (kg)			
	BMI (kg/m^2^)			
	WC (cm)			
	HOMA-IR	4.7 ± 1.6 → 3.0 ± 1.4		
	Triglyceride (mg/dL)			
	HDL (mg/dL)			

Only values where there is statistical significance between baseline and follow-up are presented. Values presented as Mean ± SD or Median (IQR)

*AHA* American Heart Association, *ALT* alanine aminotransferase, *AST* aspartate aminotransferase, *ALP* alkaline phosphatase, *BMI* body mass index, *DASH* dietary approaches to stop hypertension, *FLI* fatty liver index, *FLiO* fatty liver in obesity, *GGT* gamma-glutamyl transferase, *GSH* total glutathione, *HDL*-*C* high-density lipoprotein-cholesterol, *HOMA-IR* homoeostasis model of assessment-estimated insulin resistance, *hs-CRP* high-sensitivity C-reactive protein, *HSI* hepatic steatosis index, *LDL-C* low-density lipoprotein-cholesterol, *LF* low fat, *LFHC* low-fat high carbohydrate, *MD* Mediterranean diet, *MDA* malondialdehyde, *TAC* total antioxidant capacity, *TC* total cholesterol, *TE* transient elastography, *VLDL-C* very low-density lipoprotein-cholesterol, *WC* waist circumference

^§^Statistical significance between dietary interventions (intervention vs control) (*P* < 0.05)

^‡^Unknown statistical significance of baseline to follow-up as not reported by the authors

^*^Not statically significant, values added for comparison only

### Secondary outcomes

Weight change and BMI was assessed in six studies [23–26, 28, 29], with participants achieving significant weight loss and reduction in BMI in all but Ryan et al. (2013) [29]. However, when compared with a control group, for most studies, there was no significant difference between diets in weight loss or BMI reduction in any of the six studies [23–26, 28, 29]. Waist circumference was included in all studies as an outcome [23–29]; however, Katsagoni et al. (2018) assessed the percentage of abnormal waist circumference amongst participants [23]. All studies showed a significant reduction in waist circumference [23–29]. Within the Marin-Alejandre et al. studies, there was a significant reduction amongst all time points in the FLiO diet intervention group, compared with 6 and 12 months in the AHA control group [24, 25]. HOMA-IR was measured by all studies [23–29], with all studies showing a significant reduction, except for Properzi et al. (2018) [26]. However, when compared with a control group, all studies, other than Razavi Zade et al. (2016), showed no significant difference in HOMA-IR reduction between intervention groups [23–29].

Within the cholesterol outcomes, total cholesterol and LDL-c was assessed in six studies, all reporting a significant reduction in total cholesterol [23–28], whilst LDL-c was significantly reduced in three studies [24, 25, 28]. Marin-Alejandre et al. (2019 and 2021) showed significant reduction in total cholesterol at 6- and 12-month time points within the FLiO intervention group, and at the 6-month time point within the AHA control group, neither having a significant reduction at the 24-month time point [24, 25]. For LDL-c, the FLiO intervention group at the 12-month time point showed a significant reduction in LDL-c [25]. HDL-c was assessed in all studies, with four studies showing a significant improvement, [23, 25, 27, 28] where the 12-month time point in the FLiO intervention group within the Marin-Alejandre et al. (2021) study showed a significant improvement [25], and there was no significant difference between intervention and control in other studies [23, 24, 26–29]. For LDL-c, all studies showed no significant difference in LDL-c reduction between intervention and control [24–29], except Katsagoni et al. (2018) [23]. Triglycerides were measured in six studies [24–29], of which there was a significant reduction in five of the studies [23–28]. For the Marin-Alejandre et al. (2019 and 2021) study, at the time points 6 and 12 months, the FLiO intervention group showed a significant reduction, and at the time point of 6 months, the AHA control group showed a significant reduction in triglycerides, but at 24 months, neither the intervention nor control showed a significant reduction [24, 25]. Two studies also showed a significant difference in triglyceride reduction between intervention and control groups [27, 28].

### Certainty of evidence

The quality of outcome evidence has been summarised using the GRADE tool (Table S3). The seven reviewed studies mostly had primary and secondary outcomes that were directly comparable; however, some outcomes and measures of exposure were not. Overall, using the GRADE system, the quality of evidence was rated as Moderate. The results for each domain of GRADE for the reviewed studies follow.

#### Risk of bias

All studies were of high-quality regarding risk of bias of outcome, except for Ryan et al. 2013 and Marin-Alejandre et al. (2021) [25, 29]. All studies were randomised and included either allocation concealment or blinding. Marin-Alejandre et al. (2021) was not of high quality due to a high dropout rate, having a 59% participation rate at the 24-month time point [25]. Ryan et al. (2013) was of low quality due to a small sample size (*n* = 12) [29].

#### Inconsistency

Due to inconsistencies between outcome measures of NAFLD and types of control groups used, the seven studies could not be directly compared with each other. However, overall, all seven studies showed a similar direction of effect, with exposure to anti-inflammatory dietary patterns causing an improvement in markers of NAFLD. The common primary measure amongst all studies, ALT, showed a significant decrease in all studies except Ryan et al. (2013) [29]. Inflammatory markers studied differed but showed a similar trend where inflammatory markers significantly decreased, and anti-inflammatory markers increased.

In addition to this, the dietary pattern given to participants to adhere to in control groups varied between studies, including dietary patterns of current standard practice, macronutrient and calorie equivalents to intervention diets or ad libitum diets. In addition, for both intervention and control diets, some studies were calorie restricted.

#### Imprecision

In total, there were 334 participants across the seven reviewed studies. Due to differences in outcome measures for NAFLD, it was not possible to calculate the overall treatment effect and relative risk. It was, therefore, not possible to calculate pooled relative risk and confidence intervals, because the studies were not able to be directly compared, meaning a meta-analysis could not be conducted.

#### Indirectness

When making comparisons between studies on interventions of interest, these were indirect due to differences in NAFLD outcome measures and differences in length of dietary exposure. However, all participants studied had NAFLD, and, therefore, results are most likely generalisable to populations of people with NAFLD, other than those examined. However, in Ryan et al. (2013), it was said that the period of the intervention may have been too short to see any significant change in ALT and GGT. This was the shortest intervention period out of all seven reviewed studies [29].

All studies but one selected were comparable by their populations as the mean age was similar. Ristic-Medic et al. (2020) was the exception due to only studying males, and the mean age being lower in comparison to other studies [28].

#### Publication bias

All studies were rated high as there was no indication of missing evidence. In saying this, publication bias cannot be completely ruled out, as most studies showed significant results regarding the relationship between anti-inflammatory dietary patterns and the severity of NAFLD, but perhaps there are studies that were not published, and, therefore, not included in this review, because they had non-significant findings.

## Discussion

The present systematic review aimed to review and describe the effects of anti-inflammatory dietary patterns on NAFLD severity indices. This is the first study to systematically review the effects of anti-inflammatory dietary patterns on inflammatory markers in adults with NAFLD. A systematic review by Moosavian et al. (2020) assessed the effects of Mediterranean diet on serum metabolic profiles and anthropometric measures in adults with NAFLD, however, did not aim to assess broader anti-inflammatory dietary patterns or the effect on inflammatory markers [30]. This systematic review identified a relationship between adherence to various anti-inflammatory dietary patterns and improvement in inflammatory markers, liver enzymes, lipid profiles, insulin markers and anthropometric measures. Central to this review is the concept of inflammation contributing to NAFLD progression and the potential of an anti-inflammatory dietary pattern on mediating these pathways. Based on the findings of the seven reviewed studies, involving 255 adults with NAFLD, adherence to an anti-inflammatory dietary pattern was associated with a significant decrease in inflammatory markers, increase in anti-inflammatory markers and lower severity of NAFLD. All studies, except for Ristic-Medic et al. (2020), assessed and showed an increased adherence from baseline to end of intervention in regard to following an anti-inflammatory dietary pattern. Although a breadth of outcomes was reported, these outcomes were not evaluated across every included study. When compared to the control diet, there was no significant difference between diets for most outcomes measured. Specifically, studies assessing the inflammatory marker hs-CRP showed a significant decrease with anti-inflammatory diet adherence, compared to baseline [27, 28]. The significant decrease in hs-CRP at 8 weeks with DASH compared to control may be attributed to the intervention’s inflammatory potential, as the control diet was of macronutrient and energy equivalence [27]. Conversely, the significant decrease in hs-CRP with MD, but not when compared to LF control diet, may be related to the anti-inflammatory effects of weight loss, as a significant reduction in weight was reported in both energy-restricted diet groups [28]. Three other markers of oxidative stress were studied as they contribute to inflammation. Of these, GSH and MDA were seen to be improved significantly more than in the control group [27]. The FLiO diet was assessed within the study and shown to have similar characteristics to MD [24, 25]. At multiple time points within the study, the FLiO diet improved adiponectin levels significantly more than the AHA diet [24, 25]. Given there was no significant difference in weight loss between the intervention and control group, these improvements may be attributed to the anti-inflammatory potential of FLiO diet. The overall effect of anti-inflammatory dietary patterns was seen to be effective in most studies in improving inflammatory markers significantly more than control diets. However, as the four studies assessing inflammatory markers were also energy restricted, it is difficult to distinguish whether reduction in inflammation is related to the dietary patterns or weight loss itself. In accordance with the present results, other systematic reviews have also demonstrated that hs-CRP correlated with adiposity and that weight loss was associated with a decline in hs-CRP level, indicating weight loss may be an effective strategy for lowering levels of the inflammatory marker [31].

Anti-inflammatory dietary patterns were associated with significant improvements in most primary liver markers of the reviewed studies, suggesting a reduction in the severity of NAFLD, except for Ryan et al. (2013) [29]. The liver enzymes ALT and GGT are commonly used to reflect hepatic inflammation and injury in NAFLD patients, with ALT > 40 IU/L and GGT > 30 IU/L providing highest positive predictivity presence of NAFLD according to Sanyal et al. (2015) [32]. Reduction to levels below these ranges was not observed in the included studies. Liver stiffness outcomes in studies with longer intervention periods were significantly more effective than control groups compared with shorter intervention periods [23–26].

Similarly, FLI and HSI algorithms which utilise ALT, AST, GGT, triglyceride and BMI measures and serve as surrogate markers for liver fat were significantly improved with longer intervention periods of 12 and 24 months, but only in some shorter intervention periods [24, 25, 28]. The studies conducted by Marin-Alejandre et al. (2019, 2021) uniquely measured outcomes at 6, 12 and 24 months in subjects with NAFLD and provide valuable insight into the longer term effects of dietary intervention on patients with NAFLD [24, 25]. These findings suggest longer term anti-inflammatory dietary interventions may offer prolonged improvement in relevant diagnostic markers. Prior studies have noted the importance of adequate intervention length, with Saeed et al. (2019) suggesting a 6-month-based intervention period to provide meaningful data [33].

Most secondary outcomes showed significant improvements, most consistently for anthropometric measures, apart from Ryan et al. (2013) [29]. Importantly the intervention period used by Ryan et al. (2013) was the shortest in length and had the smallest sample size which contributed to “some concerns” for risk of bias and scoring “low” and “very low” for primary outcome measurements using the GRADE tool. Improvements in secondary outcomes reveal improvement in NAFLD risk factors of dyslipidaemia, insulin resistance and obesity. These findings suggest that whilst anti-inflammatory dietary patterns caused significant improvements in anthropometric measures, glycaemic control, and lipid profiles in comparison to control groups, there was no consistently shown benefit to adhering to one anti-inflammatory dietary pattern over other healthy eating patterns. Adults with diagnosed NAFLD tend to follow dietary patterns including high fat and sodium with suboptimal micronutrient intake and low physical activity [34]. Hence, it is unsurprising that the results show a significant improvement in secondary outcomes with both intervention and control diets as each represents a significant change to usual dietary intake.

Overweight and obesity are risk factors associated with NAFLD, with reduction in weight commonly recommended in primary care [35]. Previous studies have demonstrated the association between weight loss and improvements in NAFLD. Given five of the seven included studies implemented an energy restriction aiding in weight loss [23–25, 27, 28], it is expected that a significant reduction in weight, BMI and waist circumference was reported. In addition, the health benefits associated with these reductions includes improvements to many of the outcomes analysed in the current study. These results reflect those of Kenneally et al. (2017) who also found that 5–10% weight loss using dietary restriction, as achieved with many of the included studies, demonstrated significant reduction in steatosis and markers of NAFLD activity [36].

In studies conducted by Ristic-Medic et al. (2020) and Properzi et al. (2018) intervention periods and control diets were the same; however, the former was energy restricted, and the latter was not [26, 28]. It was seen in an energy-restricted study that all primary and secondary outcomes were significantly improved, when compared to baseline [28]. In the study that was not energy-restricted, only three of five primary outcomes and five of eight secondary outcomes significantly improved, when compared to baseline [26]. In a recent review conducted by Reddy et al. (2019), it was found that more favourable change in inflammatory markers was found with anti-inflammatory dietary interventions that were energy restricted, in adults with NAFLD [37]. In addition, more favourable change was also seen in anti-inflammatory dietary interventions as part of a co-intervention, specifically along with nutraceuticals or a pharmacological supplementation, rather than dietary intervention alone [37]. Furthermore, significant improvements in inflammatory markers were seen to be attributed to weight loss when adhering to anti-inflammatory dietary patterns, considering that significant reductions in adipocytes and IL-6 is likely to be responsible for significant reductions in CRP [37]. Similarly, a review by Soltani et al. (2018) revealed DASH diet adherence to have significant benefits on hs-CRP levels, in comparison to unhealthy or habitual diets, mediated by diet-induced weight loss [12]. When comparing the DASH diet to other healthy diets, there was no significant difference in the reducing effect on hs-CRP [12]. The anti-inflammatory dietary patterns assessed within this review, the DASH and Mediterranean diets, have both been shown to be associated with weight loss maintenance [38, 39]. It is important to note following anti-inflammatory dietary patterns has been shown to protect and prevent people from chronic conditions which are associated with inflammation, for example, reducing risk of fatal and non-fatal cardiovascular diseases by 20 percent [40]. In addition, anti-inflammatory nutrition has been shown to be associated with lower odds of NAFLD for individuals with metabolic syndrome, a risk factor for NAFLD [1, 40]. Energy-restricted dietary patterns leading to significant weight loss meant that the true effect of the anti-inflammatory diet alone, and the mechanism through which NAFLD severity significantly improved, was unclear.

### Limitations and strengths

There are limitations to this systematic review which should be considered. Significant heterogeneity was present between the included studies due to a broad range of outcomes, duration of intervention, different methods for assessment of NAFLD severity, different dietary pattern definitions, and comparator diets used. Therefore, results could not be pooled for a meta-analysis. Caloric restriction in some but not all interventions and control groups [24, 25, 27, 28], meant that any improvement in NAFLD severity or inflammatory markers could not be solely attributed to the anti-inflammatory dietary pattern. Another aspect that may also have contributed to the difficulty in specifically quantifying the effect of anti-inflammatory diets was the Hawthorne effect [41], where participants may have engaged in healthier lifestyle and dietary habits as they knew they were being studied. Of the seven included studies, only three measured inflammatory markers as outcomes as such the ability to analyse the effect of dietary interventions on inflammatory markers has been compromised. Further limitations include the limited number of included studies which matched inclusion criteria, mostly small sample sizes of participants, and the lack of effect of sex measurements. There were also strengths to the present systematic review. Registration with PROSPERO allowed for transparency of the systematic review reporting methods. The comprehensive search strategy exhausted four databases and one register. Quality assessment tools allowed for risk-of-bias assessment at the outcome level for randomised-controlled trials using RoB 2.0. Certainty of evidence for each dietary pattern and outcome was assessed through GRADE. The included randomised-controlled trials were conducted in different countries, and thus differences in participant lifestyle were considered.

## Conclusion

Anti-inflammatory dietary patterns significantly improved NAFLD severity indices, liver enzymes, lipid profiles, anthropometric measures, and glycaemic indices amongst adults with NAFLD. Significant reductions in body weight reported with both intervention and control diets may have contributed to improvements in the evaluated inflammatory and hepatic parameters. Inconsistencies amongst included studies have made it challenging to comparatively assess interventions and determine if the effect is greater than that of comparator dietary patterns, which also showed benefit, significantly reducing primary outcomes pertaining to NAFLD severity, to a similar extent. In line with this, in most of the studies, significant changes were also observed in the control group with no significant differences to the intervention group. This needs to be addressed in more depth. However, when comparing adherence to an anti-inflammatory diet to a calorie-restricted dietary pattern, an anti-inflammatory dietary pattern showed significant benefit to NAFLD severity indices. The current body of evidence of the effects of anti-inflammatory dietary patterns on NAFLD is small; nonetheless, it appears to be an effective management option for patients. The limited body of evidence indicates a requirement for further research to confirm these findings at a higher level of certainty, using isocaloric dietary patterns, longer intervention periods and studying a broader range of inflammatory markers on an adequate sample size. In addition, studies including normal-weight subjects would be valuable in identifying differences in effective nutrition therapies for this demography. Further work may help identify and more accurately assess the direct effect of anti-inflammatory dietary patterns for treatment of NAFLD and to inform future guidelines.

## Supplementary Information

Below is the link to the electronic supplementary material.

Supplementary file1 (DOCX 80 KB)

## Data Availability

The data that support the findings of this study are available upon request from the corresponding author [VH].
